# Noninvasive Monitoring of Traumatic Intracranial Hematoma Progression Using the Infrascanner: Preliminary Experience

**DOI:** 10.1016/j.acepjo.2025.100091

**Published:** 2025-03-05

**Authors:** Jan O. Jansen, Elizabeth Liptrap, Jonathan Black, Shannon W. Stephens, Timothy Smith, Hasan Ayaz, Meltem Izzetoglu, Lindy Reynolds, Russell L. Griffin, Joshua Richman, Alex Valadka, John B. Holcomb

**Affiliations:** 1 Center for Injury Science, University of Alabama at Birmingham, Birmingham, Alabama, USA; 2 Department of Neurosurgery, University of Alabama at Birmingham, Birmingham, Alabama, USA; 3 School of Biomedical Engineering, Science and Health Systems, Drexel University, Philadelphia, Pennsylvania, USA; 4 Department of Electrical and Computer Engineering, Villanova University, Villanova, Pennsylvania, USA; 5 Department of Neurological Surgery, University of Texas Southwestern Medical Center, Dallas, Texas, USA

**Keywords:** trauma, traumatic brain injury, intracranial hematoma, infrascan

## Abstract

**Objectives:**

Early detection of intracranial hematoma (ICH) expansion is critical to improving outcomes in patients with traumatic brain injuries (TBIs). The Infrascanner 2000 (InfraScan Inc) is a US Food and Drug Administration–cleared device capable of detecting ICHs. We report our preliminary experience of conducting a prospective evaluation of serial infrascans to evaluate the diagnostic performance of the device for monitoring changes in ICH size.

**Methods:**

A single-center prospective observational study was conducted. We included patients with traumatic ICHs detected on admission computed tomography (CT) scanning, and conducted hourly infrascans until a second CT scan had been performed. We evaluated the practicability of enrollment, conducting hourly infrascans, and the diagnostic performance of the device.

**Results:**

We approached 134 patients, or their legally authorized representatives, and enrolled 62 (46%). Most index hematomas were small (median, 2.5 mL, IQR, 0.6-7.0 mL), and 23 experienced enlargement. Hourly infrascan assessments were performed successfully in the majority of patients (300 of 340 scans, 88%). The most common reasons for scans not being performed, or not performed on time, were technical issues with the devices (17 scans, 40%), the presence of dressings and bandages (2 scans, 5%), and patients being taken for other investigations or treatment (12 scans, 31%). Given the sample size, the small size of enrolled patients’ ICHs, and the low proportion that experienced enlargement, it was not possible to demonstrate the ability of the Infrascanner to detect expansion.

**Conclusion:**

This preliminary study confirms the practicability of conducting a prospective evaluation of the Infrascanner for the purpose of serially monitoring the size of ICHs in trauma patients.

**The Bottom Line**

Early detection of intracranial hematoma expansion is critical to improving outcomes in patients with traumatic brain injuries. The Infrascanner 2000 (InfraScan Inc) is a US Food and Drug Administration–cleared device capable of detecting intracranial hematomas. We report our preliminary experience of conducting a prospective evaluation of serial infrascans to evaluate the diagnostic performance of the device for monitoring changes in intracranial hematoma size. The study confirms the practicability of conducting a prospective evaluation. The study was not designed, and did not demonstrate, the ability of the Infrascanner to detect expansion.

## 1 Introduction

### 1.1 Background

Traumatic brain injury (TBI) is responsible for up to half of all trauma deaths,^1^ and is a major cause of morbidity and long-term disability.^2^ In the United States, approximately 2.8 million people experience a TBI every year, resulting in 2.5 million hospital visits, 288,000 hospitalizations, and 56,800 deaths.^3^^,^^4^ The early detection of the presence and subsequent expansion of traumatic intracranial hematomas (ICHs) is vital to decreasing mortality and improving functional outcomes and quality of life.^2^^,^^5^

### 1.2 Importance

ICH expansion often occurs in the first few hours after the inciting event.^6^^,^^7^ Patients with an initially small ICH may have a relatively normal neurologic examination on admission and then deteriorate as the hematoma expands.^8^ These patients have worse outcomes than those who do not have an expanding hematoma.^5^^,^^9^ The current standard of care is in-hospital observation with serial neurologic examinations and, when indicated, repeat computed tomography (CT) scanning—both planned, or emergent when patients deteriorate. Conventionally, neurologic checks are performed every 1 to 2 hours, depending on the severity of the initial bleed.^10^^,^^11^ If no decompensation has occurred after 6 to 12 hours, the patient may undergo a follow-up CT.^10^ Patients with normal neurologic examination (mild TBI) will then be discharged home (unless they have other injuries).^12^ This process consumes considerable nursing and patient care time and frequently requires at least 2 CT scans.

**Figure 1:**
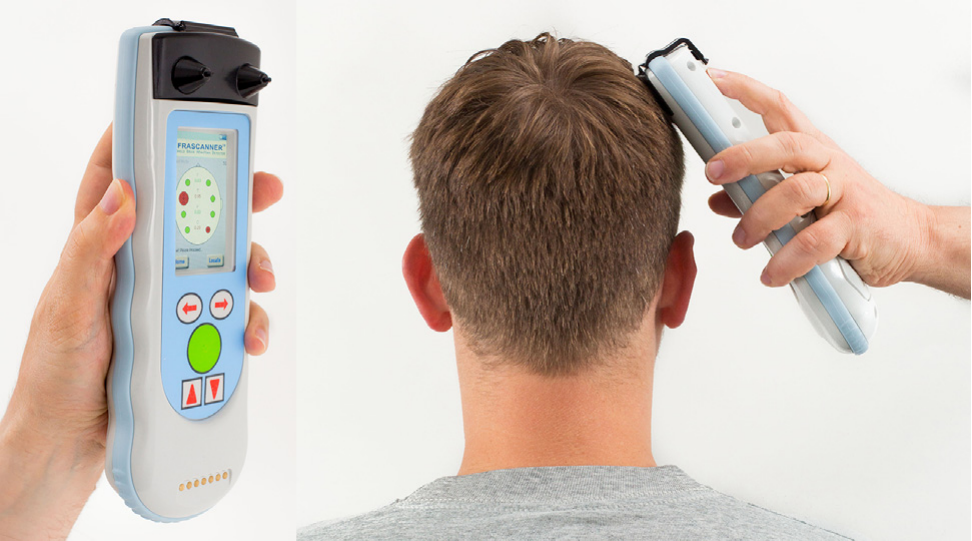
Infrascanner.

The Infrascanner 2000 (InfraScan Inc) (Fig 1) is a hand-held, noninvasive, point-of-care testing device, US Food and Drug Administration cleared to detect ICHs >3.5 mL in size, and up to 2.5 cm deep.^13^, ^14^, ^15^, ^16^ It uses near infrared (NIR)-based spectroscopy to detect increased concentrations of hemoglobin.^15^, ^16^, ^17^ Each evaluation entails a total of 8 measurements, comparing the left and right sides of the brain in 4 specific regions: frontal, temporal, parietal, and occipital (Fig 1). The device is user-friendly, battery-powered, and has good diagnostic performance. A total of 12 published studies have evaluated the diagnostic performance of the device, in terms of *detecting* ICHs.^17^ However, it may also be possible to use the Infrascanner for *monitoring* (rather than detection) previously diagnosed ICHs by conducting serial scans. Such a strategy could be used to (1) detect enlargement of ICHs before clinical deterioration occurs or (2) exclude enlargement, potentially negating the need for a second CT scan.

### 1.3 Goals of This Investigation

The purpose of this single-center pilot study was to evaluate the practicability of conducting a prospective study of serial infrascans in patients with TBI with confirmed ICHs.

## 2 Methods

### 2.1 Design

This was a prospective, observational, single institution study conducted at the University of Alabama at Birmingham (UAB), in Birmingham. The study was approved by the local IRB (300004583) as a nonsignificant risk study, with an initial waiver when patients were deemed to not have capacity, and legally authorized representatives were not available.

### 2.2 Setting

UAB is an urban, level 1 trauma center that admits approximately 4500 trauma patients annually.

### 2.3 Selection of Subjects

We included patients aged 15 years and older, with mild and moderate TBIs (defined as the presence of a definitive ICH on a CT scan, and an admission Glasgow Coma Scale [GCS] of 9 or more), and selected for nonoperative neurosurgical management. Patients had to be be enrolled within 2 hours of the radiologist’s read of the CT scan becoming available. We excluded patients expected to require operative treatment during the study period (which would have made it difficult to conduct hourly clinical examinations or infrascans), as determined by liaison with the attending trauma surgeon, and consulting subspecialties; those exhibiting cardiovascular instability; intubated patients; and known prisoners. Patients were identified by research assistants, using an enrollment form, on completion of the reads of the admission CT scans. The University of California, San Diego Brief Assessment of Capacity to Consent (UBACC) instrument^18^^,^^19^ was used to determine whether a patient had decision-making capacity. Written informed consent was then obtained from the patient or legally authorized representative. We enrolled patients on weekdays, between the hours of 7 am and 11 pm.

### 2.4 Intervention

Enrolled patients received standard neurosurgical and trauma care, including hourly neurologic assessments by the clinical team.^11^ Following each of these clinical assessments, an Infrascanner assessment was completed by a research assistant. The device does not require shaving of the patient’s head, as the coupled NIR diode laser and optical detector, covered by a disposable shield, are contained in 2 fine prongs, which can be insinuated through patients’ hair (Fig 1). The device requires the user to complete the scans in a preassigned sequence, and provides visual prompts to do so. Research assistants were trained on the use of the device by InfraScan Inc. representatives during their initial orientation training, and quarterly throughout the enrollment period thereafter. Each training session included a didactic training session and successful completion of 3 mock enrollments. Each training session lasted approximately 60 minutes. The infrascans were performed every hour (±15 minutes), until the time of the second scheduled CT scan. If a scan was not completed, not completed on time, or only partially completed, the reasons were recorded. The device does not require calibration.

The Infrascanner output available to the operator comprises 2 measures: First, the device uses binary indicators when a hematoma is present in any of the 8 measurement areas. These binary indicators signal when the optical density (OD) threshold difference has been exceeded. Second, the device also provides the “delta value,” which is a measure of the asymmetry in light attenuation (OD) between left and right sides of the head. As skull structure, brain tissue, and skin are symmetrical, the only potential source of light attenuation asymmetry is intracranial bleeding. Additional data, including the absolute ODs for each of the 8 measurement areas, are available when the Infrascanner is connected to a computer, but are not displayed to clinicians on the device. For the purpose of this study, “enlargement” (as detected by the Infrascanner) was defined as the occurrence of a new indicator demonstrating the presence of a hematoma, as compared with the previous scan. Eg, if the previous scan showed 1 of 8 positive binary indicators, and the current scan showed an additional indicator as being positive, then the latter examination was deemed to have demonstrated an enlargement.

The second CT scan was the “gold standard” against which the diagnostic performance of the Infrascanner was evaluated. Hematoma volume was calculated using the ABC/2 technique.^20^^,^^21^ An increase of more than 20% in hematoma volume was considered an enlargement.^21^

We planned to enroll up to 70 patients. As this was a pilot study, the sample size was based on convenience rather than an assumed effect size.

### 2.5 Outcomes

The outcomes of interest were (1) the practicability of enrollment (in terms of the proportion of patients or legally authorized representatives who agreed to participate, of those approached); (2) the practicability of conducting hourly infrascans in patients with TBI; and (3) the diagnostic performance of the Infrascanner in monitoring ICH size.

### 2.6 Data Analysis

All data collected were entered into a Research Electronic Data Capture (REDCap) database designed for this study. We analyzed the data by comparing each hour’s infrascan results with the previous hour’s, or the index infrascan, using 2 methods:

(1) We used the device’s binary indicator function. A positive result corresponds to an OD difference of >0.2 (or <–0.2), compared with the contralateral side, and indicates the presence of a hematoma. We evaluated the data sequentially to determine whether there was a new positive binary indicator compared with the previous scan.
(2) We used the actual OD difference displayed. For the purpose of this study, we defined an enlargement when a score in any region had >0.2 in OD difference, whereas the previous scan was not.

## 3 Results

### 3.1 Practicability of Enrollment

Over the duration of the study period (July 2020 to February 2022), we approached 134 patients who met the entry criteria, or their legally authorized representatives, and 62 (42%) agreed to participate. The median time to enrollment was 75 minutes from time of CT report (IQR, 68-106 minutes). One patient withdrew from the study after the first infrascan.

### 3.2 Characteristics of Patients Enrolled

The baseline characteristics of the study population are shown in Table 1. The median age was 67 years (IQR, 57-76). A total of 34% arrived directly from the scene, and 66% were transferred from another hospital. A total of 56% of patients were male, and the majority were White (90%). All patients had suffered blunt injuries, and the most common cause was a fall (60%). A total of 32% of patients were receiving aspirin, 3% clopidogrel, 3% warfarin, 6% rivaroxaban, 13% apixaban, and 2% dabigatran therapy at the time of injury. The median injury severity score was 14 (IQR, 10-18), and the median Abbreviated Injury Scale (AIS) head 3 (IQR 2-3). Median admission GCS was 15, with 23% of patients having a GCS < 15. Patients had, on average, normal lactate levels and base deficits, normal hemoglobin levels and platelet counts, and normal markers of coagulation. (Not all patients had all tests done, as they were not mandated by the study, and left to clinicians’ discretion.) Only 19% had capacity to consent (UBACC score >15).

**Table 1:** Baseline characteristics of study population.

Characteristic	
Demographics	
Age, years, median (IQR)	67 (57-76)
Male, n (%)	35 (56)
Race/ethnicity	
White/non-Hispanic/non-Latino, n (%)	56 (90)
American Indian/Alaskan Native/Aboriginal, n (%)	0 (0)
Asian, n (%)	0 (0)
Black/African/American, n (%)	5 (8)
Hispanic/Latino, n (%)	1 (2)
Native Hawaiian/Other Pacific Islander, n (%)	0 (0)
Other, n (%)	0 (0)
Unknown, n (%)	0 (0)
Preinjury medication	
Aspirin, n (%)	20 (32)
Clopidogrel, n (%)	2 (3)
Warfarin, n (%)	2 (3)
Rivaroxaban, n (%)	4 (6)
Apixaban, n (%)	8 (13)
Dabigatran, n (%)	1 (2)
Mechanism	
Motor vehicle collision, n (%)	16 (26)
Motor cycle collision, n (%)	5 (8)
Fall, n (%)	37 (60)
Assault, n (%)	0 (0)
Pedestrian struck by vehicle, n (%)	0 (0)
Bicycle, n (%)	0 (0)
Other, n (%)	4 (6)
Source	
Scene, n (%)	21 (34)
Transfer, n (%)	41 (66)
Injury severity and pattern	
ISS, median (IQR)	14 (10-18)
AIS head, median (IQR)	3 (2-3)
Vital signs	
GCS, median (IQR)	15 (15-15)
GCS <15, n (%)	14 (23)
SBP, median (IQR)	148 (126-162)
Admission blood work	
Lactate, mmol/L, median (IQR)	1.6 (1.2-2.2)
Base deficit, mmol/L, median (IQR)	0.3 (–1.3 to 2.5)
Hemoglobin, g/L, median (IQR)	12.5 (11.6-14.1)
Platelet count, median (IQR)	220 (173-292)
Prothrombin time, median (IQR)	13.7 (13.2-14.6)
INR, median (IQR)	1.1 (1.0-1.1)
Activated partial thromboplastin time, median (IQR)	27 (25-30)
Decision making capacity	
Yes, n (%)	12 (19)
No, n (%)	50 (81)
Admitted to ICU^a^	
Yes, n (%)	32 (52)
No, n (%)	29 (47)
Disposition^a^	
Discharged from acute care, n (%)	61 (100%)
Died, n (%)	0 (0%)
Interval between CT scans	
Second CT not performed (n)	7
Hours to second CT, median (IQR)	8.4 (6.3-14.1)
No. of infrascans attempted and/or performed	
Median per patient (IQR)	5 (4-5)
0, n (%)	1 (2)
1, n (%)	2 (3)
2, n (%)	2 (3)
3, n (%)	8 (13)
4, n (%)	12 (19)
5, n (%)	10 (16)
6, n (%)	3 (5)
7, n (%)	2 (3)
8, n (%)	20 (32)
>8, n (%)	2 (3)

AIS, Abbreviated Injury Scale; CT, computed tomography; GCS, Glasgow Coma Scale; ICU, intensive care unit; INR, international normalized ratio; ISS, Injury Severity Score; SBP, systolic blood pressure.

a Not available for one patient, who withdrew from the study.

The characteristics of the ICHs on initial CT are shown in Table 2. One patient’s CT images could not be retrieved from the server following initial review. A total of 18% of patients had an intraparenchymal hematoma, 44% a subarachnoid hematoma, and 61% a subdural hematoma on the initial CT scan. (Some patients had more than one type of hematoma.) The median total ICH volume on the initial CT scan was 2.5 mL (IQR, 0.6-7 mL). A total of 56 patients had a second CT scan. The median time between the admission and follow-up CT scans was 8.4 hours. All follow-up CTs were performed as planned investigations, rather than in response to a change in neurologic examination. The median total ICH volume on the second CT scan was 2.6 mL (IQR, 0.7-9.6 mL). The median change in volume was 0 mL (IQR, –0.9 to 1.6 mL). A total of 23 patients experienced an enlargement of their ICH. The median increase in volume for those patients who experienced enlargement was 2.5 mL (IQR, 0.4-7.5 mL). Of these patients, 13 experienced an enlargement of >20%.

**Table 2:** Hematoma characteristics on CT.

	Admission CT	Second CT
Type		
IPH, n (%)	11 (18)	10 (17)
SAH, n (%)	27 (44)	27 (46)
EDH, n (%)	0 (0)	0 (0)
SDH, n (%)	37 (61)	34 (58)
Other, n (%)	1 (2)	2 (3)
Volume		
mL, median (IQR)	2.5 (0.6-7)	2.6 (0.7-9.6)
≤1.0 mL, n (%)	19 (31)	19 (32)
1.1-2.0 mL, n (%)	7 (11)	7 (12)
2.1-3.0 mL, n (%)	9 (15)	4 (7)
3.1-4.0 mL, n (%)	4 (7)	7 (12)
4.1-5.0 mL, n (%)	3 (5)	2 (3)
5.1-6.0 mL, n (%)	2 (3)	1 (2)
6.1-7.0 mL, n (%)	2 (3)	2 (3)
7.1-8.0 mL, n (%)	0 (0)	0 (0)
8.1-9.0 mL, n (%)	2 (3)	1 (2)
9.1-10.0 mL, n (%)	0 (0)	1 (2)
>10 mL, n (%)	13 (22)	15 (25)

CT, computed tomography; EDH, extradural hematoma; IPH, intraparenchymal hematoma; SAH, subarachnoid hematoma; SDH, subdural hematoma.

Some patients had more than one type of intracranial hematoma.

None of the patients required intracranial pressure monitoring, or neurosurgical intervention. A total of 52% were admitted to intensive care unit, for a median of 3 days (IQR, 1-5). All 61 patients survived to hospital discharge. The median length of stay was 3 days (IQR, 2-7).

### 3.3 Practicability of Conducting Hourly Infrascans

The median number of infrascans performed per patient was 5. The distribution is shown in Table 1. In total, 340 infrascans were attempted. A total of 300 scans (88%) were completed (220, 64%) or partially completed (80, 24%). A total of 41 scans (12%) could not be completed. The reasons for not being able to perform scans were device problems (40%), the patient having been taken for additional imaging (not related to their TBI, 26%), and miscellaneous (staffing issues, patients being transferred between units, 27%). Scans which were incomplete (not all 8 measurements completed) were mostly due to failure to complete the occipital part of the scan due to the presence of a cervical collar (Table 3).

**Table 3:** Infrascans attempted/performed.

	n (%)
Attempted scans	340 (100)
Completed scans	220 (64)
Unable to perform scan	41 (12)
Incomplete scan	80 (24)
Reasons for inability to perform scans	
Bandages around head	2 (5)
Device problems	17 (40)
Clinical concerns about patient’s condition	0 (0)
Receiving nursing care	0 (0)
Bedside procedure being performed	2 (5)
Taken for imaging	10 (26)
Taken to operating room or interventional radiology suite	0 (0)
Other	10 (27)
Reasons for inability to perform complete scans	
Bandages around head	0 (0)
Device problems	5 (6)
Clinical concerns about patient’s condition	0 (0)
Receiving nursing care	1 (1)
Bedside procedure being performed	0 (0)
Taken for imaging	0 (0)
Taken to operating room or interventional radiology suite	0 (0)
Other	74 (93)

### 3.4 Diagnostic Performance of the Infrascanner

#### 3.4.1 Diagnostic performance for detection of ICH

The index infrascan confirmed an ICH (as shown on CT) in 48 of 61 of patients, yielding a sensitivity of 79% (95% CI, 66%-88%). Specificity could not be calculated as all included patients had an ICH.

#### 3.4.2 Diagnostic performance for detecting enlargement of ICH

These 2 analysis methods (binary indicator function and actual OD displayed) yielded similar results, but in a few instances they did not agree. These were largely cases where the optical difference shifted both magnitude and direction. For example, a score in the frontal region could shift from –0.25 to 0.25 which would remove the warning light from one side and shift it to the other (new warning light) but would not result in a new OD alert because both scans had a magnitude >0.25.

**Figure 2:**
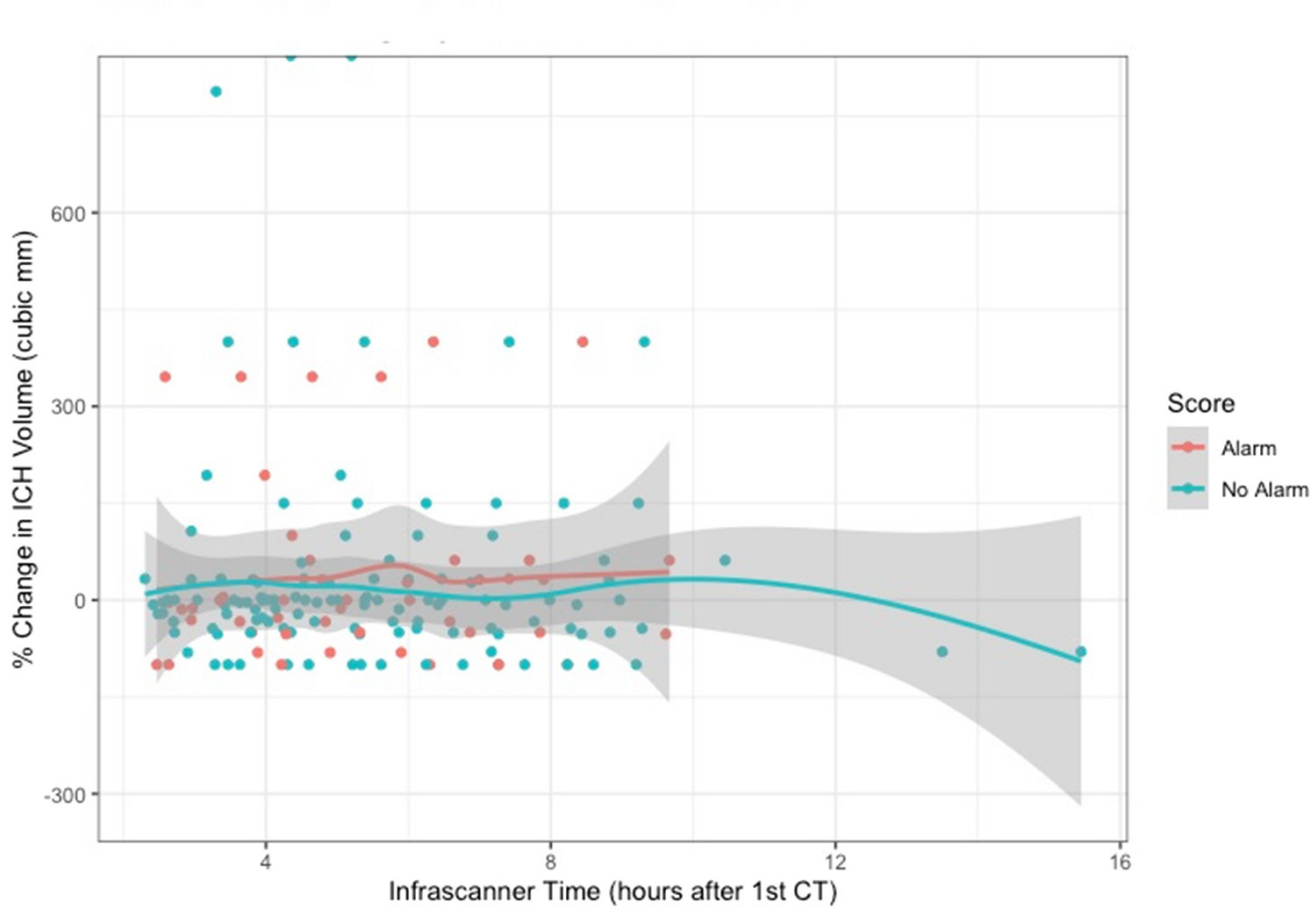
Diagnostic performance of the Infrascanner, using difference in optical density. Each point represents an infrascan where the x-axis shows elapsed time in hours since the first CT scan, and the y-axis indicates the change in volume of the intracranial hematoma, in percent, between the first and second CT scans. This change could be negative (ICH smaller on second CT scan) or positive (ICH larger on second CT scan). Because each patient only had 2 CT scans (not hourly CT scans), the change in volume does not alter over time, and the markers form a line. However, each point is color-coded by whether the infrascan at that time indicated a new warning of expansion, based on the optical density. (Red marker = Infrascanner detected expansion; green marker = Infrascanner did not detect expansion). The curves represent a nonparametric smoothing of the mean CT volume change stratified by the score alert at each time point along with confidence regions (represented by shading). Informally, separation between the curves at a time point would indicate that the infrascan sore alert at that time could be a predictor of expansion. CT, computed tomography; ICH, intracranial hematoma.

Figure 2 shows the CT volume percentage change with reference to an increase crossing the threshold of >0.2 in the OD differential. Each point represents an infrascan where the x-axis shows elapsed time in hours since the first CT scan, and the y-axis indicates the change in volume of the ICH, in percent, between the first and second CT scans. This change could be negative (ICH smaller on second CT scan) or positive (ICH larger on second CT scan). Each point is color-coded by whether the infrascan at that time indicated a warning of expansion, based on a change in OD.

Note that because each patient only had 2 CT scans, at the beginning and end of the study period, the location of the markers on the y-axis did not change over time. Red markers indicate a worse score (crossing the threshold of >0.2, indicative of hematoma enlargement) than on the previous scan, and blue markers a change in the score of <0.2. Similarly, the red line and blue lines represent the average of these readings, over time, with CIs shown as shaded areas.

Conceptually, red markers above “0,” and a red line separates from, and above, the blue line, would provide data to support that the infrascans warnings are meaningfully related to the size of the expansion assessed at the second CT scan. However, Figure 2 shows that most of the readings are clustered around the “0” line, indicating that few of the included patients experienced enlargements of their ICHs. Furthermore, the enlargements that were present were small (median, 2.5 mL). Similarly, there is no clear pattern with regard to infrascans readings that indicated an increase in ICH size (red marker) or not (blue marker).

**Figure 3:**
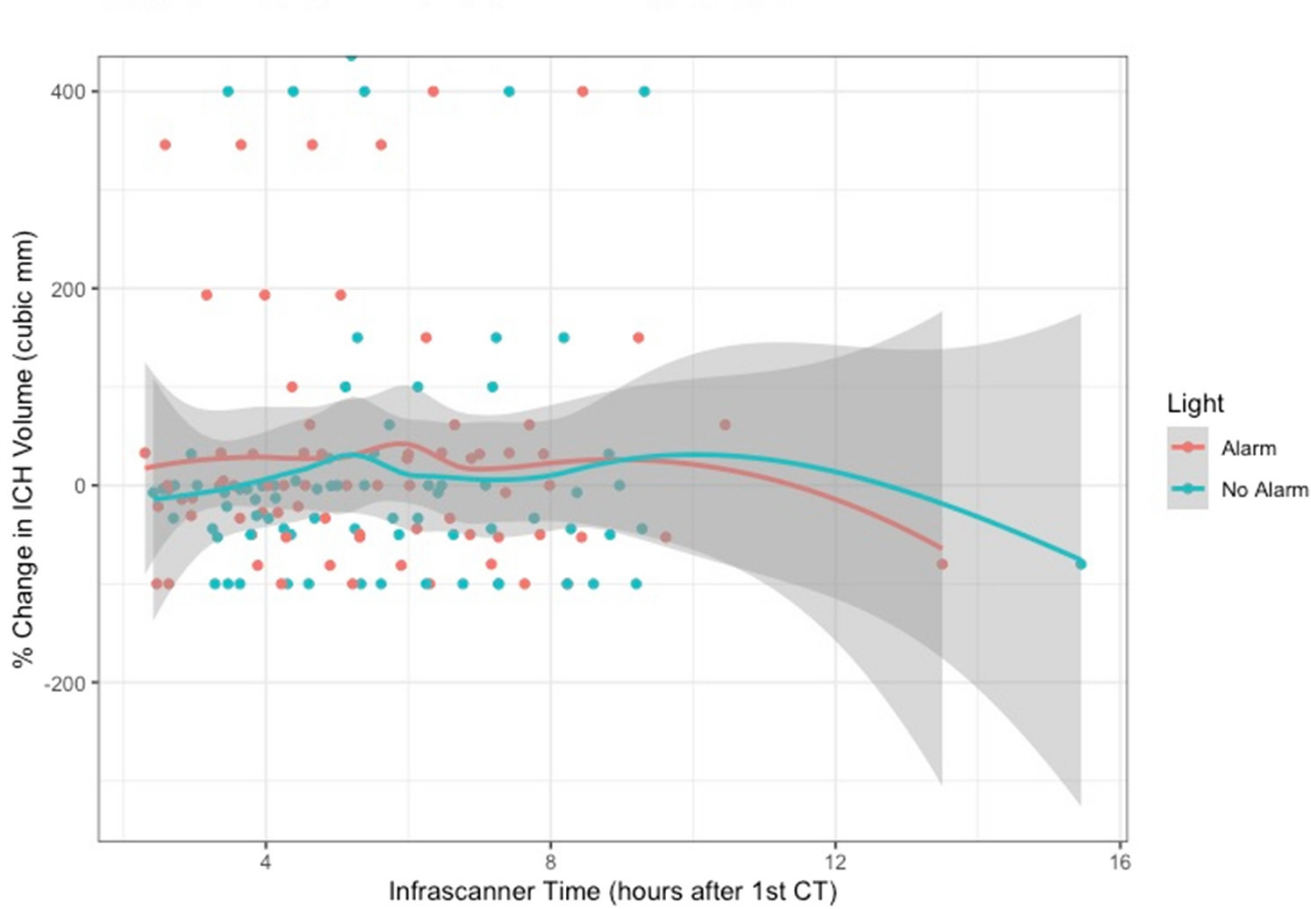
Diagnostic performance of the Infrascanner, using the binary indicator feature. Each point represents an infrascan where the x-axis shows elapsed time in hours since the first CT scan, and the y-axis indicates the change in volume of the intracranial hematoma, in percent, between the first and second CT scans. This change could be negative (ICH smaller on second CT scan) or positive (ICH larger on second CT scan). Because each patient only had 2 CT scans (not hourly CT scans), the change in volume does not alter over time, and the markers form a line. However, each point is color-coded by whether the infrascan at that time indicated a warning of expansion, based on the appearance of a new warning (red light) in a brain region that did not have a warning in the prior scan. (Red marker = Infrascanner detected expansion; green marker = Infrascanner did not detect expansion). The curves represent a nonparametric smoothing of the mean CT volume change stratified by the score alert at each time point along with confidence regions (represented by shading). Informally, separation between the curves at a time point would indicate that the infrascan sore alert at that time could be a predictor of expansion. CT, computed tomography; ICH, intracranial hematoma.

Figure 3 shows a similar graph, but this time using the Infrascanner’s built-in binary indicator alert to determine whether a scan showed enlargement, compared with the previous hour’s scan. Again, each point represents an infrascan where the x-axis shows elapsed time in hours since the first CT scan, and the y-axis indicates the change in volume of the ICH, in percent, between the first and second CT scans. As mentioned previously, this change could be negative (ICH smaller on second CT scan) or positive (ICH larger on second CT scan). Each point is color-coded by whether the infrascan at that time indicated a warning of expansion, based on the appearance of a new positive binary indicator not present on the prior scan. The appearances are similar to those seen in Figure 2.

## 4 Limitations

This study has limitations. It was a pilot study, reporting on our preliminary experience. The sample size was therefore small, and the focus was on practicability, rather than power. This was, furthermore, a single-arm study, which was not designed to detect differences in clinically meaningful outcomes. A major limitation of any evaluation of this kind is that it is not possible—for practical and ethical reasons—to conduct hourly CT scans, to produce paired results, and to directly compare the performance of the Infrascanner with CT. Precise determination of the point at which a hematoma enlarged using the gold standard test (CT scanning) is therefore not possibly. Instead, clinicians must rely on physical examination, which may vary significantly. This temporal component complicates the analysis and interpretation of the data. In addition, most patients had small ICHs. It is conceivable that the device is more likely to miss expansion in smaller bleeds than in larger hematomas. Lastly, we intentionally included patients on anticoagulants, which may impact the proportion that experienced ICH enlargement. However, therapeutic anticoagulation is becoming increasingly common, and the inclusion of such patients is therefore desirable.

## 5 Discussion

This single-center pilot study confirms the practicability of conducting a prospective evaluation of the Infrascanner for the purpose of monitoring the size of ICHs. Around half of patients or legally authorized representatives approached agreed to participate in such a study. This proportion is typical for emergency care studies requiring prospective consent. The UBACC instrument proved useful in deciding which patients had the capacity to provide consent for themselves, and which required a legally authorized representative. It was also easily administered.

Our pilot study also provides useful information regarding the inclusion and exclusion criteria for further evaluations of this kind. The criteria for this study were intentionally chosen to select patients with mild or moderate TBIs. Our results show that, although patients had a median AIS head of 3—indicating a serious brain injury—the ICHs associated with these criteria tended to be small, often below the detection threshold (3.5 mL) of the Infrascanner. Although nearly one-half of patients experienced enlargement, the absolute increase in volume was small. Future studies should consider more focused criteria, and possibly stratification of enrollment, in order to ensure that the included patients have ICHs that are large enough to be detected and enlarge. This issue impacts on the evaluation of the diagnostic performance of the Infrascanner.

This study does demonstrate the practicability of performing hourly infrascans. Nevertheless, there are useful lessons that were learned. There were some technical, device-related issues, such as failure to charge, and availability of sensor covers, which were quickly resolved in conjunction with the manufacturer. However, there were also some patient-related issues. The most important was inability to obtain occipital scans in the presence of certain types of cervical collars. It is probable that many of the issues encountered would be resolved with increasing experience of using the device.

This pilot study was not designed to evaluate the performance of the device, but to demonstrate the practicability of such an evaluation. Nevertheless, it is useful to examine the diagnostic performance data obtained. However, the findings must therefore be interpreted with caution—particularly given the small sample, and the small size of the ICHs—and the conclusions which can be drawn are thus limited. Our data demonstrate a lower sensitivity for the initial detection of ICHs than previously reported. This is, most likely, related to the small size of the hematomas (2.5 mL on the initial scan, below the approved detection limit of 3.5 mL). With regard to the monitoring of ICH size—the principal aim of the study—our results show that the variability in the sequential readings obtained is greater than anticipated. It is not clear whether this is related to the user of the device, or the device itself.

The expansion of an ICH is a serious event because it is associated with increased mortality and morbidity. However, at present, detecting such events is largely based on serial neurologic examinations, and repeat CT scanning—both planned, or emergent when patients deteriorate. The Infrascanner is easy to use, noninvasive, and does not rely on ionizing radiation, and using it to monitor hematoma size, through serial scans, is therefore highly attractive. The potential value of the device, if validated, is therefore enormous. However, a rigorous evaluation is essential. This study is a first step in this process.

In conclusion, our preliminary experience confirms the practicability of conducting a prospective evaluation of the Infrascanner for the purpose of monitoring the size of ICHs in trauma patients.

## Author Contributions

JOJ, EL, SWS, HA, MI, RL, JR, AV, and JBH designed the study. JOJ and JBH obtained funding. JOJ, EL, SWS, TS, and ELT collected the data. JOJ and JR analyzed the data. JOJ wrote the first draft of the paper. All authors reviewed and edited the manuscript.

## Funding and Support

The study was funded by a grant from InfraScan Inc. The role of the funder was to provide the Infrascanner devices, and to train the research assistants.

## Conflict of Interest

JOJ has received grants from National Institutes of Health, Department of Defense, and NIHR. He is a consultant for CSL Behring, Cellphire, and Infrascan and has received study support from CSL Behring, and Prytime Medical. SWS is a consultant for CSL Behring, and Infrascan, and has received study support from RevMedx. JBH is a consultant with Cellphire, Hemostatics, BioGenerator, Thornhill Medical, and Arsenal; is cofounder, co-CEO, and on the board of directors of Decisio Health, QinFlow, Zibrio, Hemostatics, CCJ Medical, and Oxyband; and is a coinventor of the Junctional Emergency Tourniquet Tool. The remaining authors declare no conflicts of interest.
